# The relationship between ferritin and urate levels and risk of gout

**DOI:** 10.1186/s13075-018-1668-y

**Published:** 2018-08-15

**Authors:** Tahzeeb Fatima, Cushla McKinney, Tanya J. Major, Lisa K. Stamp, Nicola Dalbeth, Cory Iverson, Tony R. Merriman, Jeffrey N. Miner

**Affiliations:** 1 Department of Biochemistry, University of Otago, Dunedin, New Zealand; 2 Department of Medicine, University of Otago, Christchurch, New Zealand; 3 Department of Medicine, University of Auckland, Auckland, New Zealand; 4 Medical Scientific Affairs, Ironwood Pharmaceuticals, Cambridge, MA USA; 5 Biology, Ardea Biosciences, Inc., San Diego, CA USA

**Keywords:** Urate, Gout, Ferritin, Iron, Association, Causal, Mendelian randomization

## Abstract

**Background:**

Ferritin positively associates with serum urate and an interventional study suggests that iron has a role in triggering gout flares. The objective of this study was to further explore the relationship between iron/ferritin and urate/gout.

**Methods:**

European (100 cases, 60 controls) and Polynesian (100 cases, 60 controls) New Zealand (NZ) males and 189 US male cases and 60 male controls participated. The 10,727 participants without gout were from the Jackson Heart (JHS; African American = 1260) and NHANES III (European = 5112; African American = 4355) studies. Regression analyses were adjusted for age, sex, body mass index and C-reactive protein. To test for a causal relationship between ferritin and urate, bidirectional two-sample Mendelian randomization analysis was performed.

**Results:**

Serum ferritin positively associated with gout in NZ Polynesian (OR (per 10 ng ml^− 1^ increase) = 1.03, *p* = 1.8E–03) and US (OR = 1.11, *p* = 7.4E–06) data sets but not in NZ European (OR = 1.00, *p* = 0.84) data sets. Ferritin positively associated with urate in NZ Polynesian (β (mg dl^− 1^) = 0.014, *p* = 2.5E–04), JHS (β = 0.009, *p* = 3.2E–05) and NHANES III (European β = 0.007, *p* = 5.1E–11; African American β = 0.011, *p* = 2.1E–16) data sets but not in NZ European (β = 0.009, *p* = 0.31) or US (β = 0.041, *p* = 0.15) gout data sets. Ferritin positively associated with the frequency of gout flares in two of the gout data sets. By Mendelian randomization analysis a one standard deviation unit increase in iron and ferritin was, respectively, associated with 0.11 (*p* = 8E–04) and 0.19 mg dl^− 1^ (*p* = 2E–04) increases in serum urate. There was no evidence for a causal effect of urate on iron/ferritin.

**Conclusions:**

These data replicate the association of ferritin with serum urate. Increased ferritin levels associated with gout and flare frequency. There was evidence of a causal effect of iron and ferritin on urate.

**Electronic supplementary material:**

The online version of this article (10.1186/s13075-018-1668-y) contains supplementary material, which is available to authorized users.

## Background

Ferritin stores excess iron in a nontoxic form. Urate is a byproduct of purine catabolism in humans. In some hyperuricemic individuals, supersaturation of urate (> 0.41 mmol/L) can result in deposition of monosodium urate (MSU) crystals in the synovium with gout arising due to an innate immune response [1]. Urate acts as a chelator for iron and, in turn, iron can modulate the activity of xanthine oxidase and the production of urate [2]. Indeed, ferritin has been positively associated with serum urate in both the US National Health and Nutrition Examination Survey (NHANES) [3] and the China Health and Nutrition Survey [4] with elevation in serum urate suggested as an indicator of iron overload [5]. Iron could contribute to gouty inflammation by forming complexes with MSU crystals, stimulating oxidative stress through the generation of reactive oxygen species, and contributing to granulocyte and complement activation [6]. A decrease in gout flares following phlebotomy to attain near iron-deficient levels in hyperuricemic patients [7] is also suggestive of a role in gout.

Food is the primary source of iron, providing heme and nonheme iron through animal and plant sources, respectively. Diet is also a key source of purines. Interestingly, purine-rich foods from an animal-based diet have been associated with increased risk of recurrent gout attacks while purine-rich foods from a plant-based diet are not [8]. These observations, combined with the other observational and intervention data [3, 5, 7], are consistent with the hypothesis that iron in purine-rich foods (e.g., red meat) could be a causal factor for gout and flare frequency.

Despite a positive association of iron/ferritin with urate, no data are yet available investigating a possible causal association. The positive association between urate and iron/ferritin could result from unmeasured confounders [9, 10]. Mendelian randomization can be used to explore the causal association between an exposure and an outcome [11]. This approach exploits random assignment of alleles at conception to disentangle cause and effect in the presence of confounding. Genetic variants that predict an observational exposure are used as unconfounded proxy instruments for the exposure itself to provide evidence of causation for the outcome of interest.

In this study, we first aimed to replicate the observational association of serum ferritin with serum urate and to test for association of serum ferritin with gout and flare frequency. The second aim was to assess a possible causal relationship between iron/ferritin and urate using Mendelian randomization.

## Methods

### Biochemical analyses

#### Study participants

The NZ participants were recruited during 2006–2014 from community-based settings and primary and secondary health care. The NZ sample set comprised male NZ European (100 cases and 60 controls) and Polynesian (Māori and/or Pacific Island ancestry; 100 cases and 60 controls) individuals. All NZ gout cases were intercritical and had a clinically confirmed diagnosis of gout by the 1977 American Rheumatology Association (ARA) preliminary classification criteria [12]. New Zealand gout cases were not screened for current use of anti-inflammatory therapy. New Zealand control participants without self-reported gout were convenience sampled from the Auckland, Otago and Canterbury regions. The US group comprised a mixture of people of Latino, African American and European ancestry (189 male gout cases and 60 male controls) and serum samples were purchased from Bioreclamation Inc. (New Cassel, NY, USA). All US gout cases had crystal-proven or clinically diagnosed gout, no active acute gout at the time of sample draw, and no nonsteroidal anti-inflammatory drug or colchicine usage within 2 weeks of the sample draw. The US control group comprised sex-matched and ancestrally-matched volunteers. Individuals with a history of liver damage or disease were excluded from the NZ and US case group and US control participants were included if they had never been diagnosed with gout and were not currently taking any nonsteroidal anti-inflammatory drug or colchicine. The Lower South Ethics Committee (OTA/99/11/098) and the NZ Multi-region Ethics Committee (MEC/05/10/130) granted ethical approval for the NZ arm. In the US, recruitment of participants for the study had applicable local regulatory requirements (including Institutional Review Board approval). Written informed consent was obtained from all subjects.

Publicly available data from two cohorts were used for serum ferritin versus urate association analyses. A total of 1260 African American individuals were included from the Jackson Heart Study (JHS) [13] via the Database of Genotypes and Phenotypes (approval #834: The Genetic Basis of Gout). The second data set was The Third National Health and Nutrition Examination Survey (NHANES III) cohort (https://wwwn.cdc.gov/nchs/nhanes/nhanes3/Default.aspx) [14], comprised of 4355 African American and 5112 European individuals. Subjects who self-reported as taking any diuretic or other urate-lowering medication(s), or had kidney disease or gout, or had first-degree relatives with gout were excluded. This categorization was made to assess urate association only in non gout subjects and to minimize bias due to gout or other potential factors affecting urate levels and thus differed from the criteria applied to the previous study that used the same NHANES III data [3]. Also, in contrast to Ghio et al. [3], we analyzed the NHANES III European and African American participants separately and adjusted by C-reactive protein (CRP) levels. Additional file 1: Tables S1 and S2 report the demographic and clinical data for all of the study groups.

#### Serum biomarker measurements

Additional file 1 describes the determination of biochemical markers (serum urate, iron, transferrin, ferritin and CRP) for this study.

#### Statistical analyses

Differences in the means for intergroup and intragroup comparisons were calculated using an unpaired *t* test in R (v3.3.2). Logistic and linear regression analyses were carried out using R v3.3.2 [15] to test for an association of serum ferritin (explanatory variable) with gout (binary response variable) and urate (continuous response variable), respectively. Individuals with missing data for any variable were excluded. Adjusted odds ratios (ORs) and β estimates were obtained by including age (in years), sex, body mass index (BMI) and CRP, and the number of self-reported Polynesian grandparents for the Polynesian sample set, as covariates in the regression models. Ferritin is a marker of inflammation [16] and therefore CRP was included as a covariate in the regression models to control for the possibility that association between ferritin/iron and risk of gout and flare frequency was a consequence of elevated ferritin levels in hyperuricemia [17] and gout [18] per se owing to the increased inflammatory milieu. The urate-producing enzyme xanthine oxidase also releases iron from ferritin [19]. Therefore, the average levels of ferritin (and other iron measures) were also compared within the NZ and US gout cases by stratifying the data sets according to the usage of the xanthine oxidase inhibitor allopurinol. Odds ratios for logistic models and β estimates for linear regression models were meta-analyzed using the meta package in R (v3.3.2). A *Q* statistic was calculated to measure the heterogeneity; if the heterogeneity was significant (*p* < 0.05) the fixed-effect model was replaced with a random-effect model.

### Mendelian randomization analysis

#### Selection of instrumental variable

Mendelian randomization (MR) analysis in this study was based on publicly available summary estimate data for European individuals from the Genetics of Iron Status Consortium (*n* = 48,978) [20] and the Global Urate Genetics Consortium (*n* = 110,347) [21]. The genetic variants were selected as instruments for iron/ferritin and urate from these GWAS and *p* ≤ 5 × 10^− 8^ was set as a threshold for a variant to be a valid instrument, corresponding to an *F* statistic of 30 and considered an adequately powered instrument for MR analysis [22]. To minimize pleiotropy, a priori literature searches and eQTL (expression quantitative trait locus) data available online (https://gtexportal.org/home/) and Haploreg v4.1(https://pubs.broadinstitute.org/mammals/haploreg/haploreg.php) were used to test for association of genetic variants to be included in instruments with expression of genes implicated in iron/ferritin and urate metabolism (as appropriate)—if there was evidence for association then the genetic variant was excluded.

#### Statistical analyses

Two-sample Mendelian randomization analysis was performed using the MR-Base (Mendelian Randomization-Base) platform (www.mrbase.org) [23]. The files for exposure instruments were manually uploaded into MR-Base. To eliminate linkage disequilibrium (LD) of *r*^2^ > 0.6 between the instruments, the ‘LD clumping’ command was used. To ensure that the effect estimate of the exposure instrument corresponds to the same allele as the outcome effect, the ‘allele harmonization’ command was used. The Wald ratio method [24, 25] was used to separately calculate the Mendelian randomization estimate for each instrument. With a single instrument, the Wald ratio method is as robust to detect a causal effect as a two-stage least squares method [26, 27]. The effect estimates were combined together in multiple-instrument MR via the inverse-variance weighted method [22], while the Egger-regression method [28] was applied as a sensitivity test for a posteriori adjustment for horizontal pleiotropy within multiple-instrument Mendelian randomization. To determine whether any individual instrument was an outlier, leave-one-out permutation analysis [29] was used as a sensitivity test. A power calculation (for a continuous outcome) was performed using the online calculator for Mendelian randomization studies (https://sb452.shinyapps.io/power) [30].

## Results

Demographic and biochemical information on the study cohorts is presented in Additional file 1: Tables S1 and S2.

### Biochemical observational association analysis

#### Association with serum urate concentration

Serum ferritin concentration was positively associated with serum urate concentration in nongout African American individuals from the JHS (males β per 10 ng ml^− 1^ increase (mg dl^− 1^) = 0.007, *p* = 9.3E–03; females β = 0.014, *p* = 1.7E–03) and NHANES III (males β = 0.006, *p* = 5.1E–04; females β = 0.016, *p* = 1.8E–15) as well as Europeans (males β = 0.004, *p* = 6.6E–03; females β = 0.012, *p* = 3.4E–10) from the NHANES III study (Table 1). It was interesting to observe the consistently stronger association in females than males (2-fold to 3-fold greater effect size in females; *P*_*Q*_ by meta-analysis of males and females was 0.27 in JHS, 1E–04 in NHANES III African American and 1.5E–03 in NHANES III European). A positive association between ferritin and SU was also observed in NZ Polynesian (β = 0.014, *p* = 2.5E–04) individuals, but not in NZ European (β = 0.009, *p* = 0.31) or US (β = 0.041, *p* = 0.15) individuals from the gout control sample sets (Table 1).

**Table 1:** Association of ferritin (per 10 ng ml^− 1^ increment) with serum urate (mg dl^− 1^)

Population	*n* ^a^	β (95% CI)^b^	*p* ^b^	β (95% CI)^c^	*p* ^c^
NZ European	60	0.011 (− 0.006; 0.027)	0.24	0.009 (− 0.009; 0.027)	0.31
NZ Polynesian	60	0.015 (0.007; 0.023)	1.4E–04	0.014 (0.007; 0.022)	2.5E–04
US	60	0.048 (− 0.006; 0.102)	0.079	0.041 (− 0.015; 0.097)	0.15
JHS (males)	567	0.008 (0.002; 0.014)	9.6E–03	0.007 (0.002; 0.014)	9.3E–03
JHS (females)	693	0.023 (0.015; 0.032)	1.6E–07	0.014 (0.005; 0.022)	1.7E–03
JHS (combined)	1260	0.027 (0.023; 0.033)	1.8E–27	0.009 (0.005; 0.014)	3.2E–05
NHANES III African American (males)	1925	0.010 (0.007; 0.014)	7.2E–09	0.006 (0.003; 0.009)	5.1E–04
NHANES III African American (females)	2430	0.028 (0.027; 0.032)	1.3E–42	0.016 (0.012; 0.020)	1.8E–15
NHANES III African American (combined)	4355	0.029 (0.026; 0.031)	2.0E–97	0.011 (0.008; 0.013)	2.1E–16
NHANES III European (males)	2460	0.006 (0.003; 0.009)	1.0E–04	0.004 (0.001; 0.007)	6.6E–03
NHANES III European (females)	2652	0.019 (0.015; 0.023)	1.3E–22	0.012 (0.008; 0.015)	3.4E–10
NHANES III European (combined)	5112	0.025 (0.023; 0.028)	6.9E–84	0.007 (0.005; 0.009)	5.1E–11
Meta-analyzed data	10,907	0.025 (0.021; 0.028)	9.3E–34	0.009 (0.007; 0.011)	3.9E–32

Beta values represent change in serum urate in mg dl^− 1^ per 10 ng ml^− 1^ increase in concentration of serum ferritin

*CI* confidence interval, *NZ* New Zealand, US United States, JHS Jackson Heart Study, NHANES National Health and Nutrition Examination Survey

^a^Number of nongout (control) individuals

^b^Unadjusted

^c^Adjusted for age, body mass index, C-reactive protein and number of self-reported Polynesian grandparents in the NZ Polynesian analyses. Combined analyses are additionally adjusted for sex

#### Association with gout

The average levels of ferritin were elevated in both NZ Polynesian (*p* = 2.3E–04) and US (*p* = 6.6E–17) gout cases compared to controls, but not between NZ European gout cases and controls (*p* = 0.21) (Table 2). An increase in serum ferritin of 10 ng ml^− 1^ was associated with an increased risk of developing gout in NZ Polynesian (OR (CI) =1.03 (1.01; 1.05), *p* = 1.8E–03) and US (OR (CI) =1.11 (1.07; 1.17), *p* = 7.4E–06) individuals. However, ferritin was not associated with increased risk of gout in the NZ European sample set (OR (CI) =1.00 (0.97; 1.02), *p* = 0.84) (Table 2). Exclusion of gout patients on allopurinol treatment from the US sample set did not influence the association of ferritin levels with gout (OR (CI) =1.11 (1.06; 1.16), *p* = 2.4E–05).

**Table 2:** Comparison of average values of serum ferritin (ng ml^− 1^) in gout case–control groups and the association with gout

Population	Comparison of average		Association analysis			
	*p*	(95% CI)_for difference_	OR (95% CI)^a^	*p* ^a^	OR (95% CI)^b^	*p* ^b^
NZ European	0.21	(−97.46; 22.02)	1.01 (0.99; 1.03)	0.22	1.00 (0.97; 1.02)	0.84
NZ Polynesian	2.3E–04	(− 211.88; − 66.31)	1.03 (1.01; 1.05)	1.7E–03	1.03 (1.01; 1.06)	1.8E–03
US	6.6E–17	(− 167.19; − 107.16)	1.25 (1.07; 1.17)	1.5E–06	1.11 (1.07; 1.17)	7.4E–06
US no allopurinol	6.6E–17	(−167.19; − 107.16)	1.11 (1.07; 1.16)	4.5E–06	1.11 (1.06; 1.16)	2.4E–05
Meta-analysis^c^	–	–	1.04 (1.01; 1.08)	0.02	1.04 (0.99; 1.09)	0.09

All OR values represent change in risk per 10 ng ml^−1^ increase in serum ferritin

*CI* confidence interval, *OR* odds ratio, *NZ* New Zealand, US United States

^a^Unadjusted

^b^Adjusted for age, body mass index, C-reactive protein and number of self-reported Polynesian grandparents in the NZ Polynesian analyses

^c^Only sample sets not stratified for allopurinol exposure were included in the meta-analysis

#### Association of ferritin with flare frequency

We also tested for an association of serum ferritin, CRP and serum urate with self-reported frequency of gout flares (per year). Each 10 ng ml^− 1^ increase in ferritin was associated with an increased frequency of gout flares in the US (β = 0.02 flares/year, *p* = 2E–03) and NZ European (β = 0.09, *p* = 0.04) individuals but not in NZ Polynesian (β = − 0.11, *p* = 0.14) individuals. C-reactive protein and urate concentrations were not associated with frequency of gout flares in all three data sets (Table 3).

**Table 3:** Association of serum ferritin, CRP and serum urate with gout flares/year

Population	Ferritin (ng ml^− 1^)				C-reactive protein (mg dl^− 1^)		Serum urate (mg dl^− 1^)	
	β (95% CI)^a^	*p* ^a^	β (95% CI)^b^	*p* ^b^	β (95% CI)^a^	*p* ^a^	β (95% CI)^a^	*p* ^a^
NZ European	0.09 (0.003; 0.17)	0.042	0.069 (− 0.01; 0.14)	0.067	1.75 (− 3.85; 7.37)	0.54	− 0.61 (− 1.48; 0.25)	0.16
NZ Polynesian	− 0.11 (− 0.24; 0.03)	0.14	−0.091 (− 0.22; 0.03)	0.15	−9.95 (− 23.75; 3.84)	0.16	−1.59 (− 3.88; 0.71)	0.17
US	0.02 (0.01; 0.04)	0.002	0.018 (0.001; 0.04)	0.043	0.10 (− 0.13; 0.35)	0.38	0.13 (− 0.04; 0.30)	0.13

Values represent change in the annual frequency of flares per 10 ng ml^− 1^ increase in serum ferritin. Levels of urate are at the time of subject recruitment

*CRP* C-reactive protein, *CI* confidence interval, *NZ* New Zealand, US United States

^a^Adjusted for age, body mass index and number of self-reported Polynesian grandparents in the NZ Polynesian analyses

^b^Additionally adjusted for allopurinol usage

#### Association of ferritin levels with allopurinol use

Significantly increased serum ferritin levels were observed among participants with gout who reported taking allopurinol in the NZ Polynesian (*p* = 0.005) and US (*p* = 0.02) sample sets; however, the average levels of ferritin were not significantly different in the NZ European sample set when the same stratification was done (*p* = 0.47) (Table 4).

**Table 4:** Self-reported allopurinol use and iron profile comparison in gout patients

Population/marker	No allopurinol	Allopurinol	*p*	(95% CI)_for difference_
NZ European (*n*)	23	66	–	
Serum iron (μmol L^−1^)	100.44	98.65	0.77	(− 10.661; 14.258)
Serum ferritin (ng ml^− 1^)	253.71	284.31	0.48	(− 115.787; 54.597)
NZ Polynesian (*n*)	22	74		
Serum iron (μmol L^−1^)	91.21	85.08	0.26	(− 4.592; 16.816)
Serum ferritin (ng ml^−1^)	373.81	494.32	0.005	(− 204.466; − 36.548)
US (*n*)	155	34		
Serum ferritin (ng ml^− 1^)	158.57	248.21	0.02	(− 164.816; 14.451)

*CI* confidence interval, *NZ* New Zealand, US United States

#### Analysis with log-transformed ferritin

The ferritin distribution deviated from normality for all study groups (Additional file 1: Figure S1, S2), therefore the data were log transformed. Results for the log-transformed (normalized) data are presented in Additional file 1: Tables S3–S5. The results were similar to those from the untransformed data.

### Mendelian randomization analysis

The Mendelian randomization analysis had 100% power to detect a causal effect (change in outcome in SD units per SD change in exposure) of 0.1 at *p* < 0.05. The two GWASs selected for two-sample MR (84,978 for iron measures [20] and 110,347 for serum urate [21]) had an overlap of 20,160 individuals; this overlap is considered sufficiently low to avoid any potential bias in Mendelian randomization estimates [31].

Additional file 1: Table S6 provides the list of genetic variants evaluated as instrumental variables for iron (*n* = 3), ferritin (*n* = 5) and urate (*n* = 3). Details of selection of these instruments, along with approaches to minimize pleiotropy, are outlined in Additional file 1 and Additional file 1: Figure S3.

#### Iron biomarkers as exposures for urate as outcome

The strongest signal for iron and ferritin at *HFE rs1800562* (allele A) in the exposure data was found to causally increase urate levels in Europeans (iron β = 0.11 mg dl^− 1^ increase in serum urate per standard deviation unit increase in iron, *p =* 0.0008; ferritin β = 0.19, *p =* 0.0002) (Table 5). A causal effect of ferritin levels on urate levels was also observed when *ABO rs651007* (allele T) was used as a single instrument in the Mendelian randomization model (β = 0.32, *p =* 0.02) (Table 5). However multiple-instrument Mendelian randomization using the inverse-variance weighted method did not indicate any causal effect of iron (β = 0.056, *p* = 0.15, *P*_*Q*_ = 0.18) or ferritin (β = 0.089, *p* = 0.17, *P*_*Q*_ = 0.06) on urate (Table 5). The nonsignificant effect remained consistent after a posteriori adjustment for horizontal pleiotropy, with effects across the variants showing heterogeneity, via MR-Egger for both iron (β = 0.064, *p* = 0.61, *P*_*Q*_ = 0.01) and ferritin (β = 0.16, *p* = 0.32, *P*_*Q*_ = 0.01) (Table 5). MR-Egger did not indicate high pleiotropy for an effect on urate using all three instruments for iron (*p*_intercept_ = 0.95) and five instruments for ferritin (*p*_intercept_ = 0.58) (Table 5). However, leave-one-out sensitivity analysis indicated *TMPRSS6 rs855791* and *SLC40A1 rs12693541* to be potential outliers driving a noncausal observed effect of iron and ferritin on urate, respectively (Table 6). Leaving these two variants out in a subsequent combined inverse-variance weighted analysis demonstrated iron (β = 0.11, *p* = 1.96E–04) and ferritin (β = 0.14, *p* = 0.03) to causally increase urate levels in Europeans (Table 6).

**Table 5:** Association between iron/ferritin and urate using two-sample Mendelian randomization

MR analysis	Phenotype		Gene/locus	Genetic variant	estimate	SE	(95% CI)	*p*-causal	*Q–p*	MR Egger_HP	
	Exposure	Outcome								intercept	*p* value
Wald ratio	Iron	Urate	*TF*	*rs1525892*	0.127	0.076	(− 0.02; 0.28)	0.093	–	–	–
–	Iron	Urate	*HFE*	*rs1800562*	0.107	0.032	(0.04; 0.17)	0.0008	–	–	–
–	Iron	Urate	*TMPRSS6*	*rs855791*	− 0.001	0.031	(− 0.06; 0.06)	0.99	–	–	–
IVW	Iron	Urate	–	All	0.056	0.039	(− 0.02; 0.13)	0.15	0.179	–	–
MR Egger	Iron	Urate	–	All	0.064	0.109	(− 0.15; 0.28)	0.61	0.011	− 0.0016	0.95
Wald ratio	Log ferritin	Urate	*SLC40A1*	*rs12693541*	− 0.068	0.076	(− 0.22; 0.08)	0.37	–	–	–
–	Log ferritin	Urate	*HFE*	*rs1800562*	0.190	0.057	(0.08; 0.30)	0.0002	–	–	–
–	Log ferritin	Urate	*TMPRSS6*	*rs2413450*	0.011	0.106	(− 0.20; 0.22)	0.92	–	–	–
–	Log ferritin	Urate	*TEX14*	*rs411988*	− 0.048	0.123	(− 0.29; 0.19)	0.70	–	–	–
–	Log ferritin	Urate	*ABO*	*rs651007*	0.320	0.140	(0.05; 0.59)	0.022	–	–	–
IVW	Log ferritin	Urate	–	All	0.089	0.066	(− 0.04; 0.22)	0.18	0.063	–	–
MR Egger	Log ferritin	Urate	–	All	0.160	0.134	(− 0.10; 0.42)	0.32	0.014	− 0.0067	0.58

All beta estimates presented as an effect of per standard deviation unit change in iron and ferritin on change in urate (mg dl^−1^)

*MR* Mendelian randomization, *SE* standard error, *CI* confidence interval, p-*causal p* value using MR analysis, Q–p Cochran’s heterogeneity test showing *p* value for heterogeneity, *MR Egger_HP* Egger test for horizontal pleiotropy, *IVW* meta-analysis using inverse-variance method, *MR Egger* Mendelian randomization using Egger regression

**Table 6:** Leave-one-out sensitivity analysis for association between iron/ferritin and urate using inverse-variance weighted two-sample Mendelian randomization

Phenotype		Instrumental variant excluded from IVW analysis	β estimate	(95% CI)	*p*-causal
Exposure	Outcome				
Iron	Urate	*TF rs1525892*	0.050	(− 0.06; 0.16)	0.35
Iron	Urate	*HFE rs1800562*	0.017	(− 0.07; 0.10)	0.70
Iron	Urate	*TMPRSS6 rs855791*	0.110	(0.05; 0.17)	2.0E–04
Iron	Urate	All	0.050	(− 0.02; 0.13)	0.15
Log ferritin	Urate	*SLC40A1 rs12693541*	0.141	(0.02; 0.27)	0.027
Log ferritin	Urate	*HFE rs1800562*	0.007	(− 0.14; 0.15)	0.93
Log ferritin	Urate	*TMPRSS6 rs2413450*	0.101	(− 0.05; 0.26)	0.20
Log ferritin	Urate	*TEX14 rs411988*	0.103	(− 0.04; 0.25)	0.17
Log ferritin	Urate	*ABO rs651007*	0.070	(− 0.06; 0.20)	0.30
Log ferritin	Urate	All	0.089	(− 0.04; 0.22)	0.18

All beta estimates presented as an effect of per standard deviation unit change in iron and ferritin on change in urate (mg dl^−1^)

*IVW* meta-analysis using inverse-variance method, *CI* confidence interval, p*-causal p* value using IVW meta-analysis, *HFE* human hemochromatosis protein, *TMPRSS6* transmembrane protease serine-6, *ABO* alpha 1–3-*N*-acetylgalactosaminyltransferase

#### Urate as exposure for iron biomarkers as outcome

None of the urate instruments (*SLC2A9 rs12498742*, *SLC16A9 rs1171614* and *SLC22A12 rs478607*) indicated causality for serum urate levels on iron and ferritin levels when tested individually (Additional file 1: Table S7). The noncausal relationship remained consistent when analyzed using multiple-instrument inverse-variance weighting and a posteriori adjustment for horizontal pleiotropy using MR-Egger (Additional file 1: Table S7). By leave-one-out analysis, none of the urate instruments was an outlier for a causal effect on iron or ferritin (Additional file 1: Table S8).

## Discussion

We extended the previously reported associations of serum ferritin with serum urate [3, 4] using adjustment by CRP to exclude the possibility that the relationship is a consequence of the association of ferritin with inflammatory states, particularly since there is evidence that serum urate levels drop in acute inflammation in hospitalized patients [32]. The association of serum ferritin with serum urate has now been reported in European, Chinese, African American, and NZ Māori and Pacific (Polynesian) sample sets. We associated increased serum ferritin with the risk of gout and gout flares, although for both relationships an association was observed in only two of the three data sets used. Given that allopurinol exposure raises ferritin levels and given the limited number of gout patients not exposed to allopurinol available to us, we cannot conclude that the gout–ferritin association is independent of allopurinol exposure. Mendelian randomization single instrument analysis (Table 5) and multiple instrument leave-one-out analysis (Table 6) provided evidence for a causal role of iron and ferritin in increasing urate levels in Europeans. In single instrument analysis, *HFE rs1800562* provided evidence of causality. In multiple instrument ‘leave-one-out’ sensitivity analysis, excluding two variants (*TMPRSS6 rs855791* and *SLC40A1 rs12693541*) also provided evidence of causality. These variants may obscure a causal effect because they mediate an unidentified link between urate and ferritin metabolism.

Metal ions can potentially cause oxidative stress when bound to storage or transport proteins [33] and urate is a well-known antioxidant in humans, by scavenging oxygen radicals, singlet oxygen and oxo-heme oxidants [34–36]. Thus it has been proposed that urate could reduce iron-catalyzed oxidative stress by acting as a metal chelator [37]. Xanthine oxidase acts as the sole enzymatic source of urate in humans and exposure to iron may enhance its activity [2, 38, 39]. This may explain a causal effect of iron on serum urate levels. A Mendelian randomization analysis demonstrated causality of iron/ferritin on renal function with increased levels being protective [40]. Given that reduced renal function is causal of hyperuricemia, it is unlikely that our Mendelian randomization data, supporting a causal role for iron/ferritin increasing serum urate levels, is mediated via an effect on kidney function. An increase in urate in both rodents [41, 42] and humans [43] in response to acute exposure to iron also supports a direct link between levels of iron and urate. Elevated serum urate is sometimes used as a cue to screen for hemochromatosis [5]. Our study, however, did not provide any evidence supporting a causal effect of urate on iron/ferritin levels.

We found a positive correlation between the number of gout flares and ferritin. Iron deposits have been reported to be consistently present in the synovial membrane of people with rheumatoid arthritis but not those with other joint pathologies [44]. Some rodent studies have also demonstrated an improvement in joint-related inflammation following the removal of iron by chelation treatment [45, 46]. Maintenance of near iron deficiency by depleting the levels via phlebotomy in people with gout induces either complete or marked reduction in incidence and severity of gout flares in humans [7].

Iron-rich foods are top triggers for gout flares: 62.5% people with gout reporting seafood/fish, 35.2% red meat [47]. There is a positive correlation between the consumption of red meat and incident gout risk in a study of 28,990 men [48], and another study in 47,150 men associated the consumption of meat and seafood, but not purine-rich vegetables, with an increased risk of incident gout [49]. A Turkish retrospective study indicated that higher consumption of total meat (including fish) acts as a precipitating factor for gout flares [50]. Also, purines from animal-based, but not plant-based, food have been associated with increased risk of recurrent gout attacks [8]. In line with these findings there is an association of increased consumption of red meat with hyperuricemia in NHANES III [51]. It is possible that, additional to purines, the iron content of animal-based foods contributes to the risk of gout and flares.

There was elevation of serum ferritin levels in individuals on allopurinol treatment. This is of interest as xanthine oxidase is involved in the release of iron from ferritin and facilitating cellular stress through the production of hydroxyl radicals [19]. Use of allopurinol as a xanthine oxidase inhibitor has been attributed to an increased iron overload in rodent liver cells and elevated serum iron in patients with secondary gout [52]. Although a possible influence of allopurinol in iron metabolism in human is unclear, our results are consistent with previous reports [53, 54], namely that our data imply higher serum ferritin levels in gout patients on allopurinol therapy. Confirming the possible effect of allopurinol on iron metabolism in gout patients, using cohorts that are matched for clinical features of gout, could assist decision-making in treatment options for patients with a risk of iron overload. It is interesting to observe that a possible consequence of allopurinol exposure in gout could be an increase in serum urate levels via a possible causal effect of increased ferritin levels, although any increase would be considerably smaller than the decrease exacted by inhibition of xanthine oxidase by allopurinol.

There are several limitations to our study. Firstly, the gout cases were ascertained differently between the NZ and US cohorts (by clinical diagnosis only and by a combination of clinical diagnosis and crystal-proven gout, respectively). Furthermore, the NZ gout cases were not screened for current exposure to anti-inflammatory medication, a potential confounder. Secondly, only a limited number of possible confounders were adjusted for in the serum ferritin versus serum urate/gout/flares analyses. Other possible confounders could be alcohol (and other dietary exposures), liver steatosis and diabetes. The primary reason for using a limited set of confounders was the limited data available in the US gout sample set. Therefore, our observational epidemiological data can only support the observation that increased ferritin levels associate with serum urate levels, the risk of gout and the number of self-reported flares and cannot be used to claim that this association is independent of known confounders. Certainly, our data support a role for allopurinol in raising ferritin levels (Table 4). We do note, however, that the Mendelian randomization analysis supported a causal role for increased serum ferritin levels in increasing serum urate levels. Thirdly, self-report of gout flares can be regarded as an inaccurate method of flare frequency ascertainment, although it is the only practical way of collecting these data in large cohorts for epidemiological studies. However, it has been reported that, compared to the gold standard of physician assessment of flare, self-reported flares have a high sensitivity of 91% [55]. Finally, the Mendelian randomization data are unreplicated and only performed using cohorts of European ancestry. Currently it is not possible to replicate owing to the unavailability of suitable independent sample sets.

The consistency of our observational findings with previously reported experimental and clinical intervention studies and the positive causal association via Mendelian randomization analysis, albeit unreplicated, support the argument that there is a causal relationship between serum iron/ferritin and urate. This could have clinical implications with respect to causing gout. (We are unaware of any studies investigating links between hemochromatosis and gout.) Furthermore, our data support investigation of the possibility of an iron-rich diet as a trigger for gout flares that may ultimately improve flare avoidance advice given to people with gout.

## Conclusions

This study further confirmed the observational relationship between increased serum ferritin and iron levels and serum urate levels. We provide the first evidence for positive association of serum ferritin levels with the risk of gout and with the frequency of gout flares. By Mendelian randomization there was evidence for a causal relationship for ferritin and iron in increasing urate levels, but not for urate increasing ferritin and iron levels. Clinically, our data suggest that consideration of avoidance of iron-rich foods could improve flare avoidance advice given to people with gout.

## Additional file

Additional file 1: Supplemental methods, **Tables S1.**–**S8.** and **Figures S1.**–**S3**. (DOCX 578 kb)

## Abbreviations

ABO: Alpha 1–3-*N*-acetylgalactosaminyltransferase
ARA: American Rheumatology Association
BMI: Body mass index
CI: Confidence interval
CRP: C-reactive protein
eQTL: Expression quantitative trait locus
GWAS: Genome-wide association study
HFE: Human hemochromatosis protein
JHS: Jackson Heart Study
LD: Linkage disequilibrium
MR: Mendelian randomization
MSU: Monosodium urate
NHANES: National Health and Nutrition Examination Survey
NY: New York
NZ: New Zealand
OR: Odds ratio
SD: Standard deviation
SLC: Solute carrier
TMPRSS6: Transmembrane protease serine-6
US: United States
