# Supplementary material for: The relationship between ferritin and urate levels and risk of gout
Source: Arthritis Res Ther. 2018 Aug 15;20:179. doi: 10.1186/s13075-018-1668-y (PMC6094576; doi:10.1186/s13075-018-1668-y)
Supplement: Supplementary file 1 — Supplemental methods, Tables S1.–S8. and Figures S1.–S3. (DOCX 578 kb) [file 13075_2018_1668_MOESM1_ESM.docx]

**Additional file**

**The relationship between ferritin and urate levels and risk of gout**

Tahzeeb Fatima^1^, Cushla McKinney^1^, Tanya J Major^1^, Lisa K Stamp^2^, Nicola Dalbeth^3^, Cory Iverson^4^, Tony R Merriman^1^*, Jeffrey N Miner^4^

^1^Department of Biochemistry, University of Otago, Dunedin, New Zealand; ^2^Department of Medicine, University of Otago, Christchurch, New Zealand; ^3^Department of Medicine, University of Auckland, Auckland, New Zealand; ^4^Ardea Biosciences, Inc., San Diego, CA, USA.

*Address correspondence to Tony R. Merriman, Email: [tony.merriman@otago.ac.nz](mailto:tony.merriman@otago.ac.nz)

**Supplemental Methods**

**S 1.1 Biochemical analyses**

*Plasma/serum urate measurements*

Serum urate measurement in NZ subjects was carried out by the uricase oxidation method. The endpoint measurement was done using fully automated Roche Cobas® 8000 Modular P/D analyser and reagents provided by the manufacturers. The median coefficient of variation for this machine is 2.1% and the within-sample measurement correlation 99.6%. For the US participants, urate was measured by liquid chromatography-tandem mass spectrometry (LC-MS/MS) in a replicate plasma biomarker sample aliquot at Seventh Wave Laboratories (Missouri, USA). Separation was achieved on a Synergi Polar-RP 80A (4.6 x 50 mm, 4 µm) column with mobile phases of 0.1 % formic acid in water and 0.1 % formic acid in acetonitrile. Quantification was done using a multiple reaction-monitoring mode to monitor the precursor-to-product ion transitions of mass to charge ratio or m/z 167.0 to m/z 124.0 for urate and m/z 169 to m/z 125 for 1,3-(15) N urate in negative ionization mode. The calibration curve was established over the range of 10-250 µg/mL, and the correlation coefficient was 0.999. The accuracy determined at eight concentrations ranged between 92.7 and 107%.

*Measurement of ferritin and other iron markers and C-reactive protein*

For NZ participants, serum iron profile markers were measured using Roche Cobas^®^ systems (Roche Diagnostics GmbH D-68298 Mannheim, Germany). Standard laboratory protocols provided by Cobas^®^ systems e602, c702 and c701/702 were followed to measure total iron, ferritin and transferrin, respectively.

Total iron binding capacity (TIBC) and transferrin saturation (TSAT) was calculated;

TIBC (μmolL^-1^) = 25.1 × Transferrin (gL^-1^)

TSAT (%) = Iron / TIBC × 100

Where ‘25.1’ is the theoretical ratio of TIBC (μmolL^-1^) to transferrin (gL^-1^)

C reactive protein levels (CRP) in NZ subjects were measured using commercially available CRP human enzyme-linked immunosorbent assay (ELISA) kits from Abcam (ab99995 from R&D Systems, USA), according to the manufacturer’s instructions.

The Myriad Rules Based Medicine Human Multi Analyte Profile system (Myriad RBM, Inc., Austin, Texas; https://myriadrbm.com/) was used to measure plasma ferritin (accession number P02794 and P02792) and CRP (accession number: P02714) in the 249 US case-control subjects. An aliquot of each sample was added to individual microsphere multiplexes of the selected Multi Analyte Profile and blocker. This mixture was thoroughly mixed and incubated at room temperature for 1 hour. Multiplexed cocktails of biotinylated reporter antibodies were added robotically and after thorough mixing incubated for an additional hour at room temperature. Multiplexes were labeled using an excess of streptavidin-phycoerythrin solution, thoroughly mixed and incubated for one hour at room temperature. The volume of each multiplexed reaction was reduced by vacuum filtration and washed three times. After the final wash, the volume was increased by addition of buffer for analysis using a Luminex instrument and the resulting data interpreted using proprietary software developed by Myriad RBM. For each multiplex reaction, both calibrators and controls were included on each microtiter plate. Eight-point calibrators to form a standard curve were run in the first and last column of each plate and controls at three concentration levels were run in duplicate. Study sample values for each of the analytes were determined using four and five parameter logistics, weighted and non-weighted curve fitting algorithms included in the data analysis package.

The methodologies followed for biochemical measurements in JHS for urate ^1^, iron profile ^2^ and CRP ^3^ measurements for the JHS are described in the cited references while ref ^4^ provides details for the laboratory measurement protocols in the NHANES III cohorts.

**S 1.2 Mendelian randomization analysis**

***Methods***

*Selection of iron exposure instruments*

Of the variants associated with iron-related phenotypes in the combined discovery and replication data from the Genetics of Iron Status consortium ^5^, the *HFE* variant *rs1799945* was excluded from the list because of the possible pleiotropic effect with a known urate locus (*SLC17A1-A3*) – it is reported to be a *cis*-eQTL (eQTL: expression quantitative trait loci) for the urate-associated solute carrier family 17 member 3 gene (*SLC17A3*: *p =* 8.5E-06) in the adrenal gland ([www.gtexportal.org](http://www.gtexportal.org)) ^6^. Another iron-associated SNP within the *TFR2* gene, *rs7385804*, has been reported to be associated with the expression of solute carrier family 12 member 9 gene (*SLC12A9*: *p =* 1.29E-05) ^7^ and Ephrin type-B receptor 4 gene (*EPHB4*: *p =* 2.7E-34) in whole blood ([www.archive.broadinstitute.org](http://archive.broadinstitute.org/)). The *SLC12A9* gene encodes a protein that acts as the inhibitor of member 2 gene within the same family (*SLC12A2*) ^8^. Expressed ubiquitously, *SLC12A2* is a Na^+^-K^+^-Cl^-^ cotransporter, which is inhibited by diuretics ^9^ and its increased activity is associated with increased blood pressure in animals ^10^. As blood pressure could influence renal function and hence urate levels independent of iron metabolism, a pleiotropic effect could not be ruled out. The *EPHB4* gene is known to upregulate podocyte activity in glomeulonephritis ^11^, and possibly could have a pleiotropic effect on urate levels via an iron-independent influence on renal function ^12^. For all other genetic instruments, no evidence of a pleiotropic effect was found. The variant, *HFE*: *rs1800562,* has been described as a *cis*-eQTL for tripartite motif-containing protein 8 (*TRIM38*), histone gene cluster 1 (*HIST1H-2AC, 2BD, 4A* and *4H*) and for lincRNA of unknown function (*U91328.19* and *22*) ([www.archive.broadinstitute.org](http://archive.broadinstitute.org/); [www.gtexportal.org](http://www.gtexportal.org/)). On the basis of evidence available in the literature, *TRIM38* was found to be involved in the production of E3 ubiquitin-protein, which negatively regulates the immune response via toll-like receptors ^13^, although there is no evidence for involvement in urate metabolism or renal function ^14 15^. *Rs1800562* is in close proximity to the urate locus *SLC17A1-4* ^6^, however there is negligible linkage disequilibrium with the *SLC17A1-4* variants associated with serum urate (Fig. S3). SNP *rs651007* is located in the non-coding region of the *ABO* gene and is known to be associated with a number of traits including red blood cell count ^16^. The *ABO* gene encodes the glycosyltransferase responsible for the A-B-O blood groups. This variant has been reported to be associated with the expression of two other genes, Surfeit 6 (*SURF6*) and Globoside alpha-1,3-N-acetyl-galactossaminyl-transferase (*GBGT1*) in the *cis*-eQTL database ([www.gtexportal.org](http://www.gtexportal.org/) and [www.archive.broadinstitute.org](http://archive.broadinstitute.org/)). Based on the evidence available in the literature, *SURF6* is a member of the family of genes that codes for RNA-binding proteins in the nucleolus ^17^, while *GBGT1* not only encodes for the ABO-related glycosyltransferase but also participates in the biosynthesis of a glycolipid heterophil protein called ‘the Forssman antigen’ ^18^. The SNP *TMPRSS6: rs855791* is a missense variant that increases the enzyme efficiency for inhibiting hepcidin ^19^ and has been reported to be protective against iron deficiency anaemia in older women ^20^. The gene *TMPRSS6* (transmembrane protease serine 6) encodes a transmembrane enzyme called ‘serine 6’, and is reported to be involved in extracellular matrix remodeling within the liver ([www.ncbi.nlm.nih.gov](http://www.ncbi.nlm.nih.gov)), sensing iron deficiency and promoting its absorption via blocking the gene encoding for hepcidin ^21^. SNP *rs12693541* is a non-coding variant within *SLC40A1* (solute carrier family 40 member 1) that encodes ferroportin. Ferroportin is the only known mammalian iron exporter in duodenal and other epithelial cells. Defects in the *SLC40A1* gene can cause haemochromatosis type 1 and 4 ^22 23^ and reduced iron export ^24^. The SNP *rs12693541* has been reported as a *cis*-eQTL for two other genes; ‘asparagine synthetase domain containing 1 (*ASNSD1*) and ORMDL sphingolipid biosynthesis regulator 3 (*ORMDL3*) ([www.archive.broadinstitute.org](http://archive.broadinstitute.org/) and [www.gtexportal.org](http://www.gtexportal.org/)).

*Selection of urate exposure instruments*

The variants selected as instruments for urate exposure were exclusively selected from solute transporter genes associated with urate (*SLC2A9, SLC22A11/A12, SLC16A9*) ^6^. *ABCG2* was not included owing to evidence for a heme transport role for ABCG2 ^25^. Variants within the *SLC17A1-A3* locus were not considered given that expression of *SLC17A3* associates with *HFE rs1799945* (refer above). *SLC2A9* *rs12498742* was the lead urate-associated SNP by GWAS ^6^. No *cis*-eQTL has been reported for *rs12498742* in two gene-expression platforms used in this study (HaploRegv4: [www.archive.broadinstitute.org](http://archive.broadinstitute.org/) and GTEx portal: [www.gtexportal.org](http://www.gtexportal.org)) except *SLC2A9* in the whole blood (*p =* 6.42E-09). The second instrumental variable (*rs1171614*) was selected from solute carrier family 16 member 9 (*SLC16A9*), which encodes for MCT9 (monocarboxylate transporter 9) protein. The SNP *rs1171614* has been reported to be mainly associated with expression of only *SLC16A9* in several tissues in *cis*-eQTL data ^7^ i.e., artery aorta (*p =* 1.92E-06), oesophagous mucosa: (*p =* 2.31E-08) and thyroid gland (*p =* 1.77E-06) ([www.archive.broadinstitute.org](http://archive.broadinstitute.org/) and [www.gtexportal.org](http://www.gtexportal.org)). The third instrumental variable (*rs478607*) was selected from the gene *SLC22A12/URAT1* (solute carrier family 22 member 12) that encodes a urate transporter. The SNP *rs478607* has been reported to be a *cis*-eQTL for expression of *SLC22A12* in several tissues and expression of splicing factor 1 (*SF1*) gene in whole blood (*p =* 3.15E-08) ([www.archive.broadinstitute.org](http://archive.broadinstitute.org/) and [www.gtexportal.org](http://www.gtexportal.org)), with no suggestion of pleiotropy. No data were found for the *SLC22A11: rs2078267* variant in the outcome GWAS ^5^, which is why this variant was not included as an IV in the MR model. Also, there was no variant reported in the outcome GWAS that could be used as a proxy for *rs2078267.*

Table S1 Demographic and clinical information for study groups

| Populations | NZ European | | | NZ Polynesian  [NZ Māori and Pacific Island] | | | US  [Latino, African American and European] | | JHS  [African American] | NHANES III  [European] | NHANES III  [African American] |
| --- | --- | --- | --- | --- | --- | --- | --- | --- | --- | --- | --- |
| Group | **Non-gout** | **Gout** | | **Non-gout** | **Gout** | | **Non-gout** | **Gout** | **Non-gout** | **Non-gout** | **Non-gout** |
| Total Participants | 60 | 100 | | 60 | 100 | | 60 | 189 | 1260 | 5112 | 4355 |
| Baseline Information | | | | | | | | | | | |
| Males, number (%) | 60 (100) | 100 (100) | | 60 (100) | | 100 (100) | 60 (100) | 189 (100) | 567 (45.00) | 2460 (48.09) | 1925 (44.41) |
| Age, Mean ± SD | 53.41 ± 14.95 | 64.3 ± 11.73 | | 41.98 ± 13.71 | | 48.18 ± 13.42 | 52.1 ± 6.89 | 56.55 ± 10.76 | 47.59 ± 10.82 | 52.95 ± 19.87 | 41.90 ± 17.63 |
| BMI, Mean ± SD | 26.30 ± 3.02 | 30.05 ± 4.46 | | 31.09 ± 5.68 | | 36.32 ± 7.75 | - | - | 31.16 ± 7.23 | 26.01 ± 5.05 | 27.65 ± 6.58 |
| SU (mmolL^-1^), Mean ± SD | 0.33 ± 0.07 | 0.38 ± 0.10 | | 0.32 ± 0.03 | | 0.49 ± 0.10 | 0.35 ± 0.08 | 0.38 ± 0.11 | 0.31 ± 0.08 | 0.31 ± 0.07 | 0.32 ± 0.09 |
| CRP (mgdL^-1^), Mean ± SD | 0.39 ± 0.25 | 0.59 ± 0.28 | | 0.39 ± 0.26 | | 0.60 ± 0.27 | 0.61 ± 0.81 | 1.03 ± 1.44 | 0.43 ± 0.71 | 0.41 ± 0.62 | 0.52 ± 0.82 |
| Iron Profile Measurements | | | | | | | | | | | |
| Serum iron (μgdL^-1^), Mean ± SD | 105.74 ± 30.91 | 100.62 ± 33.76 | | 90.01 ± 29.07 | | 82.91 ± 27.77 | - | - | 81.38 ± 31.61 | 90.21 ± 36.39 | 80.18 ± 35.62 |
| Serum ferritin (ngmL^-1^), Mean ± SD | 230.71 ± 182.91 | 268.42 ± 197.04 | | 323.39 ± 173.22 | | 462.42 ± 245.25 | 69.45 ± 63.41 | 206.63 ± 176.13 | 157.89 ± 156.95 | 130.62 ± 139.86 | 144.21 ± 163.74 |
| Serum transferrin (gL^-1^), Mean ± SD | 2.52 ± 0.39 | 2.53 ± 0.34 | | 2.62 ± 0.47 | | 2.79 ± 0.57 | - | - | - | - | - |
| TIBC (μmolL^-1^), Mean ± SD | 63.47 ± 9.99 | 63.65 ± 8.76 | | 65.82 ± 12.01 | | 70.18 ± 14.40 | - | - | 52.85 ± 8.91 | 62.70 ± 10.01 | 62.14 ± 10.54 |
| TSAT (%), Mean ± SD | 30.22 ± 9.12 | 28.8 ± 10.06 | | 24.92 ± 8.05 | | 21.56 ± 7.11 | - | - | 28.13 ± 11.05 | 26.37 ± 11.36 | 23.72 ± 10.81 |
| Gout Characteristics | | | | | | | | | | | |
| No of gout flares/year, Mean ± SD | - | | 3.72 ± 7.02 | - | | 7.73 ± 23.71 | - | 2.02 ± 1.87 | - | - | - |
| % Allopurinol treatment (% reported) | - | | 74.16 (89) | - | | 77.08 (96) | - | 17.98 (100) | - | - | - |
| % Gout tophus (% reported) | - | | 27 (100) | - | | 66 (100) | - | NA | - | - | - |

Table S2 Detailed demographic and clinical information for non-gout individuals from JHS and NHANES III study groups: stratified on the basis of gender

| Populations | JHS [African American] | | | NHANES III [European] | | | NHANES III [African American] | |
| --- | --- | --- | --- | --- | --- | --- | --- | --- |
| Group | **Males** | **Females** | | **Males** | **Females** | | **Males** | **Females** |
| Number (%of total) | 567 (45.00) | 693 (55.00) | | 2460 (48.09) | 2652 (51.87) | | 1925 (44.41) | 2430 (55.79) |
| Baseline Information | | | | | | | | |
| Age, Mean ± SD | 47.57 ± 10.63 | 47.61 ± 10.98 | | 54.22 ± 19.54 | | 51.77 ± 20.10 | 42.04 ± 17.67 | 41.79 ± 17.61 |
| BMI, Mean ± SD | 29.57 ± 6.03 | 32.47 ± 7.85 | | 26.34 ± 4.39 | | 25.70 ± 5.57 | 26.32 ± 5.22 | 28.71 ± 7.31 |
| SU (mmolL^-1^), Mean ± SD | 0.34 ± 0.07 | 0.26 ± 0.07 | | 0.35 ± 0.07 | | 0.27 ± 0.06 | 0.36 ± 0.08 | 0.28 ± 0.08 |
| CRP (mgdL^-1^), Mean ± SD | 0.29 ± 0.49 | 0.55 ± 0.82 | | 0.39 ± 0.63 | | 0.42 ± 0.61 | 0.42 ± 0.67 | 0.60 ± 0.92 |
| Iron Profile Measurements | | | | | | | | |
| Serum iron (μgdL^-1^), Mean ± SD | 90.22 ± 30.23 | 74.14 ± 30.87 | | 94.92 ± 35.14 | | 85.83 ± 36.98 | 89.89 ± 35.11 | 72.48 ± 34.13 |
| Serum ferritin (ngmL^-1^), Mean ± SD | 224.47 ± 180.17 | 103.38 ± 107.95 | | 178.81 ± 152.05 | | 85.97 ± 110.121 | 204.03 ± 175.96 | 96.77 ± 135.80 |
| TIBC (μmolL^-1^), Mean ± SD | 51.02 ± 7.01 | 54.58 ± 9.32 | | 60.91 ± 9.02 | | 64.37 ± 10.58 | 60.03 ± 9.24 | 63.81 ± 11.19 |
| TSAT (%), Mean ± SD | 31.96 ± 10.52 | | 24.99 ± 10.48 | 28.41 ± 11.17 | | 24.49 ± 11.21 | 27.18 ± 10.57 | 20.98 ± 10.19 |

**Table S3 Association of log ferritin (ngmL^-1^) with serum urate (mgdL^-1^)**

| Population | ß [95% CI] | *P* | ß [95% CI]* | *P** |
| --- | --- | --- | --- | --- |
| NZ European | 0.366 [-0.041 ; 0.773] | 0.076 | 0.334 [-0.103 ; 0.770] | 0.13 |
| NZ Polynesian | 0.367 [0.124 ; 0.619] | 3.4E-03 | 0.380 [0.135 ; 0.626] | 2.5E-03 |
| US | 0.383 [-0.065 ; 0.831] | 0.092 | 0.322 [-0.155 ; 0.799] | 0.18 |
| JHS (males) | 0.182 [0.044 ; 0.320] | 9.8E-03 | 0.158 [0.024 ; 0.292] | 2.1E-02 |
| JHS (females) | 0.291 [0.198 ; 0.385] | 1.6E-09 | 0.159 [0.063 ; 0.255] | 1.2E-03 |
| JHS (combined) | 0.525 [0.451 ; 0.599] | 7.2E-41 | 0.181 [0.104 ; 0.258] | 4.1E-06 |
| NHANES III European (Males) | 0.144 [0.087 ; 0.202] | 8.9E-07 | 0.085 [0.030 ; 0.140] | 2.4E-03 |
| NHANES III European (Females) | 0.297 [0.256 ; 0.338] | 9.3E-44 | 0.193 [0.151 ; 0.234] | 2.1E-19 |
| NHANES III European (Combined) | 0.479 [0.446 ; 0.513] | 6.1E-163 | 0.177 [0.144 ; 0.211] | 1.8E-25 |
| NHANES III African American (Males) | 0.231 [0.157 ; 0.303] | 7.9E-10 | 0.123 [0.052 ; 0.194] | 6.7E-04 |
| NHANES III African American (Females) | 0.376 [0.331 ; 0.422] | 2.6E-56 | 0.209 [0.161 ; 0.257] | 1.9E-17 |
| NHANES III African American (Combined) | 0.523 [0.486 ; 0.559] | 4.5E-160 | 0.206 [0.167 ; 0.245] | 1.5E-24 |

*Adjusted for age, sex, BMI, C-reactive protein and number of self-reported Polynesian grandparents in the NZ Polynesian analyses. All ß-estimates represent a change per unit log of ferritin.

**Table S4 Association of Log ferritin with gout**

| Population | OR [95% CI] | *P* | OR [95% CI]* | *P** |
| --- | --- | --- | --- | --- |
| NZ European | 1.19 [0.81 ; 1.76] | 0.37 | 0.88 [0.49 ; 1.52] | 0.64 |
| NZ Polynesian | 1.76 [1.12 ; 2.84] | 1.6E-02 | 2.24 [1.27 ; 4.17] | 7.4E-03 |
| US | 2.42 [1.79 ; 3.39] | 7.0E-08 | 2.31 [1.65 ; 3.31] | 2.3E-06 |

*Adjusted for age, sex, BMI, C-reactive protein and number of self-reported Polynesian grandparents in the NZ Polynesian analyses. All odds ratios represent a change per unit log of ferritin.

**Table S5 Association of Log ferritin (ngmL^-1^) with gout flares (per year)**

| Population | ß [95% CI] | *P* | ß [95% CI]* | *P** |
| --- | --- | --- | --- | --- |
| NZ European | 1.74 [0.17 ; 3.32] | 0.030 | 1.94 [0.24 ; 3.63] | 0.025 |
| NZ Polynesian | -4.42 [-9.75 ; 0.90] | 0.10 | -4.68 [-10.11 ; 0.75] | 0.090 |
| US | 0.38 [0.11 ; 0.64] | 0.0063 | 0.37 [0.10 ; 0.63] | 0.0066 |

*Adjusted for age, sex, BMI and number of self-reported Polynesian grandparents in the NZ Polynesian analyses. All ß-estimates represent a change per unit log of ferritin.

Table S6 Details for the exposure-associated instrument variants selected for the study

| Expo/Outc phenotype | rs ID | Allele | | Chr | Gene/Nearest gene | Exp-GWAS_ß | SE | Exp-GWAS_*p* | OutC-GWAS_*p* | *r^2^*  (in LD with) | eQTL-gene (*p*)/ pleiotropy/Status |
| --- | --- | --- | --- | --- | --- | --- | --- | --- | --- | --- | --- |
|  |  | **Eff** | **Ref** |  |  |  |  |  |  |  |  |
| Iron profile-related instruments | | | | | | | | | | | |
| Iron/Urate | *rs1525892* | A | G | 3 | *TF* | 0.074 | 0.0104 | 1.7E-12 | 0.096 | - | - |
|  | *rs1800562* | A | G | 6 | *HFE* | 0.372 | 0.02 | 4.0E-77 | 0.001 | - | - |
|  | *rs855791* | A | G | 22 | *TMPRSS6* | -0.186 | 0.0101 | 4.3E-77 | 0.98 | - | - |
|  | *rs1799945* | C | G | 6 | *HFE* | -0.189 | 0.01 | 1.1E-81 | 2.9E-06 | - | *SLC17A3* (8.5E-06) |
|  | *rs7385804* | A | C | 7 | *TFR2* | 0.064 | 0.007 | 1.4E-18 | 0.40 | - | Pleiotropic |
|  | *rs8177240* | T | G | 3 | *TF* | -0.089 | 0.011 | 2.4E-12 | 0.091 | 1 (*rs1525892*) | - |
| Ferritin/Urate | *rs12693541* | T | C | 2 | *SLC40A1* | -0.106 | 0.014 | 4.2E-14 | 0.38 | - | - |
|  | *rs1800562* | A | G | 6 | *HFE* | 0.211 | 0.0187 | 1.4E-29 | 0.001 | - | - |
|  | *rs2413450* | T | C | 22 | *TMPRSS6* | -0.056 | 0.0095 | 3.6E-09 | 0.91 | - | - |
|  | *rs368243* | T | C | 17 | *TEX14* | 0.051 | 0.0093 | 3.8E-08 | 0.62 | 0.9 (*rs411988*) | - |
|  | *rs651007* | T | C | 9 | *ABO* | -0.05 | 0.009 | 1.3E-08 | 0.021 | - | - |
|  | *rs411988* | A | G | 17 | *TEX14* | -0.044 | 0.007 | 1.6E-10 | 0.69 | - | - |
|  | *rs1799945* | C | G | 6 | *HFE* | -0.065 | 0.01 | 1.4E-18 | 2.9E-06 |  | *SLC17A3* (8.5E-06) |
| Urate-related instruments | | | | | | | | | | | |
| Urate/Iron | *rs12498742* | A | G | 4 | *SLC2A9* | 0.373 | 0.006 | 1E-700 | 0.31 | - | - |
|  | *rs1171614* | T | C | 10 | *SLC16A9* | -0.079 | 0.007 | 2.3E-28 | 0.94 | - | - |
|  | *rs2078267* | T | C | 11 | *SLC22A11* | -0.073 | 0.006 | 9.4E-38 | - | - | Not in outcome data |
|  | *rs478607* | A | G | 11 | *SLC22A12* | -0.047 | 0.007 | 4.4E-11 | 0.87 | - | - |
| Urate/Ferritin | *rs12498742* | A | G | 4 | *SLC2A9* | 0.373 | 0.006 | 1E-700 | 0.46 | - | - |
|  | *rs1171614* | T | C | 10 | *SLC16A9* | -0.079 | 0.007 | 2.3E-28 | 0.55 | - | - |
|  | *rs2078267* | T | C | 11 | *SLC22A11* | -0.073 | 0.006 | 9.4E-38 | - | - | Not in outcome data |
|  | *rs478607* | A | G | 11 | *SLC22A12* | -0.047 | 0.007 | 4.4E-11 | 0.059 | - | - |

Ferritin: Log ferritin, Exp/Outc: Exposure and outcome traits, Eff: effect allele, Ref: reference allele, Chr: chromosome, SE: standard error, Exp-GWAS*_*ß: Effect estimate for association with exposure trait in Exposure (presented as SD units for iron, ferritin, transferrin and TSAT and as mgdL^-1^ for urate) GWAS, Exp-GWAS*_p*: *p*-value from exposure association GWAS, OutC-GWAS*_p*: *p*-value from outcome association GWAS, *r^2^*: R-squared value for the LD (linkage disequilibrium) with another SNP in the list, eQTL-gene: gene with which the said SNP is co-expressed, eQTL: expression quantitative trait loci, Status: Absence of selected IV in the outcome data.

Table S7 Association between urate and iron-related traits using two-sample Mendelian randomization

| MR-analysis Method | Phenotype | | Gene/locus | Instrument variant | ß-estimate | SE | [95% CI] | *p*-causal | *Q-p* | MR Egger_HP | |
| --- | --- | --- | --- | --- | --- | --- | --- | --- | --- | --- | --- |
|  | **Exposure** | **Outcome** |  |  |  |  |  |  |  | **intercept** | ***p*-value** |
| Wald ratio | Urate | Iron | *SLC2A9* | *rs12498742* | -0.032 | 0.031 | [-0.09 ; 0.03] | 0.31 | - | - | - |
| - | Urate | Iron | *SLC16A9* | *rs1171614* | 0.011 | 0.158 | [-0.30 ; 0.32] | 0.94 | - | - | - |
| - | Urate | Iron | *SLC22A12* | *rs478607* | 0.046 | 0.291 | [-0.52 ; 0.62] | 0.87 | - | - | - |
| IVW | Urate | Iron | *-* | All | -0.029 | 0.031 | [-0.09 ; 0.03] | 0.34 | 0.97 | - | - |
| MR Egger | Urate | Iron | - | All | -0.043 | 0.047 | [-0.14 ; 0.05] | 0.53 | 1.00 | 0.0042 | 0.772 |
| Wald ratio | Urate | Log Ferritin | *SLC2A9* | *rs12498742* | 0.022 | 0.029 | [-0.04 ; 0.08] | 0.46 | - | - | - |
| - | Urate | Log Ferritin | *SLC16A9* | *rs1171614* | 0.091 | 0.152 | [-0.21 ; 0.39] | 0.55 | - | - | - |
| - | Urate | Log Ferritin | *SLC22A12* | *rs478607* | -0.519 | 0.274 | [-1.06 ; 0.02] | 0.058 | - | - | - |
| IVW | Urate | Log Ferritin | - | All | 0.018 | 0.041 | [-0.06 ; 0.10] | 0.66 | 0.36 | - | - |
| MR Egger | Urate | Log Ferritin | - | All | 0.057 | 0.077 | [-0.09 ; 0.21] | 0.60 | 0.089 | -0.0118 | 0.634 |

All - IVW: Meta-analysis using inverse-variance method, All - MR Egger: Mendelian randomization using Egger regression, ß: Beta estimates, SE: Standard error, 95% CI: 95% confidence interval, *p*-causal: *p*-value using MR analysis, *Q-p*: Cochran’s heterogeneity test *p*-value for heterogeneity. All ß-estimates are presented as an effect of per mgdL^-1^ change in urate for per SD unit change in iron biomarkers.

Table S8 Results of leave-one-out sensitivity analysis for association between urate and iron-related traits using two-sample Mendelian randomization

| Phenotype | | Instrument variant excluded from IVW analysis | ß-estimate | [95% CI] | *p*-causal |
| --- | --- | --- | --- | --- | --- |
| Exposure | **Outcome** |  |  |  |  |
| Urate | Iron | *rs12498742* | 0.094 | [-0.25 ; 0.29] | 0.89 |
| Urate | Iron | *rs1171614* | -0.031 | [-0.09 ; 0.03] | 0.32 |
| Urate | Iron | *rs478607* | -0.030 | [-0.09 ; 0.03] | 0.32 |
| Urate | Iron | All | -0.029 | [-0.09 ; 0.03] | 0.34 |
| Urate | Log Ferritin | *rs12498742* | -0.052 | [-0.56 ; 0.45] | 0.84 |
| Urate | Log Ferritin | *rs1171614* | 0.015 | [-0.10 ; 0.13] | 0.79 |
| Urate | Log Ferritin | *rs478607* | 0.024 | [-0.03 ; 0.08] | 0.40 |
| Urate | Log Ferritin | All | 0.018 | [-0.06 ; 0.10] | 0.66 |

IVW: meta-analysis using inverse-variance method, ß: beta estimate, 95% CI: 95% confidence interval, *p*-causal: *p*-value using IVW meta-analysis. All ß-estimates are presented as an effect of per mgdL^-1^ change in urate for per SD unit change in iron biomarkers.

**
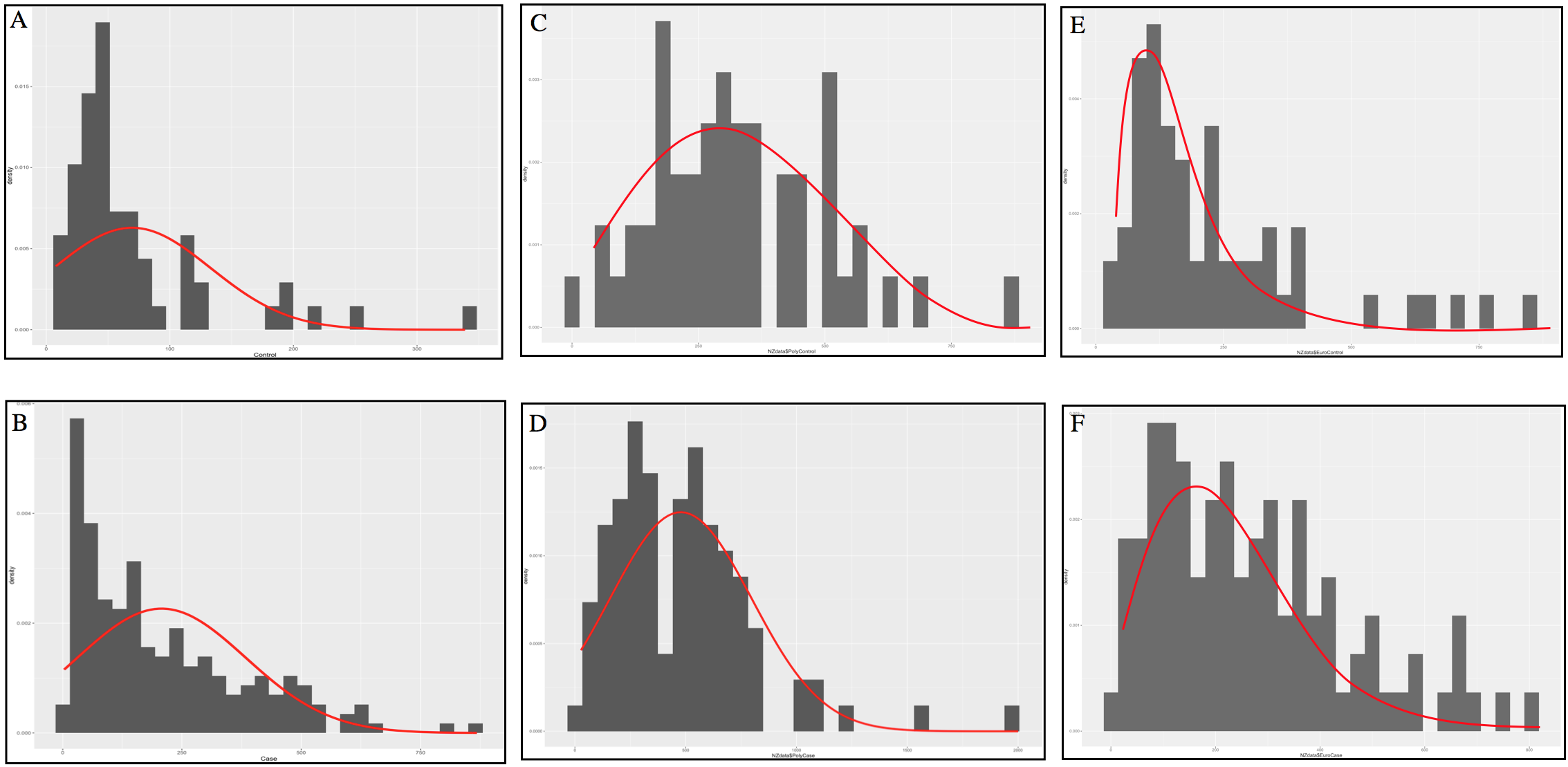
Figure S1:** Histograms indicating non-normal distribution of ferritin data in US (A) control and (B) case, NZ Polynesian (C) control and (D) case and NZ European (E) control and (F) case groups.

A


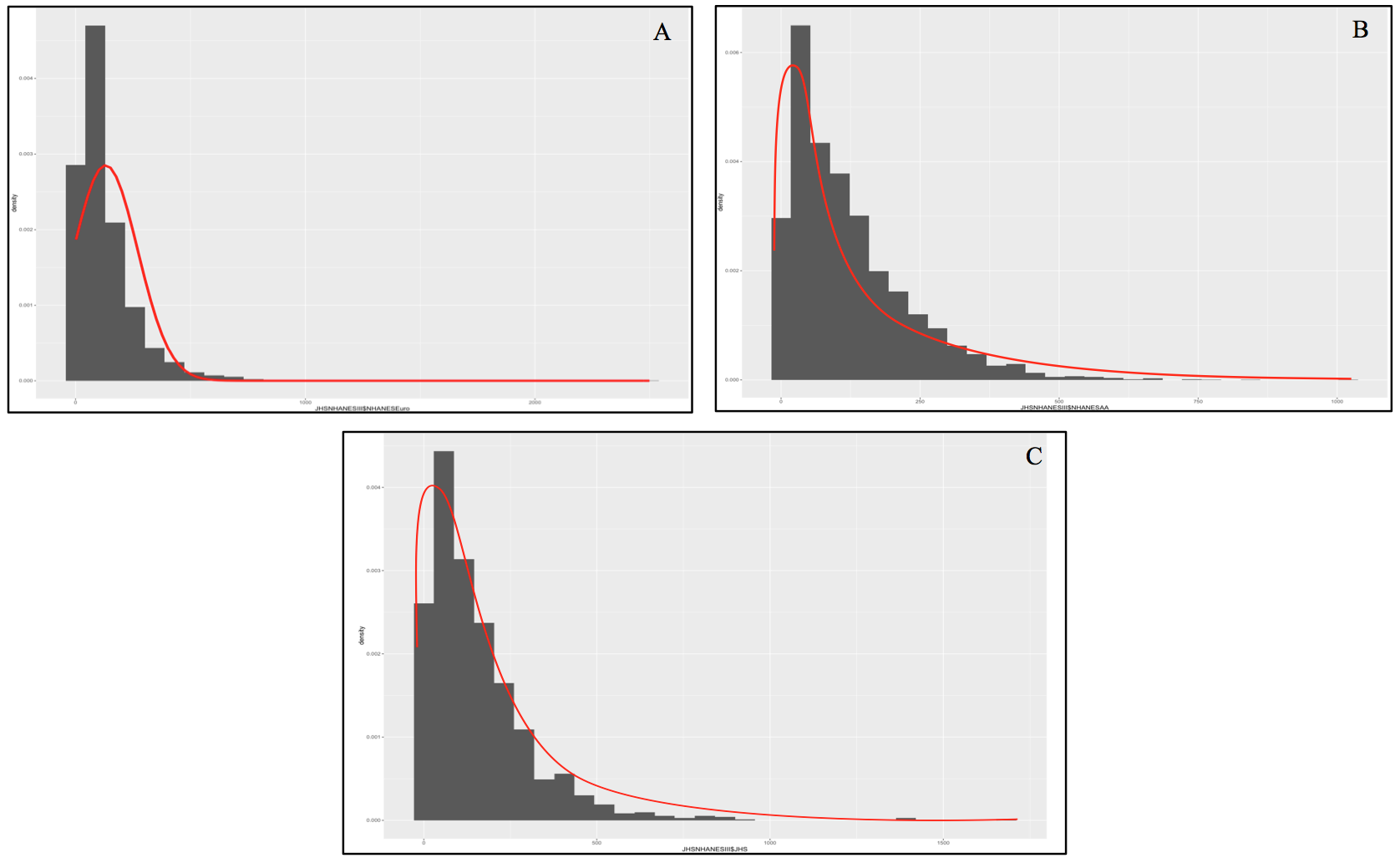


**Figure S2**: Histograms indicating non-normal distribution of ferritin data in (A) NHANES III European, (B) NHANES III African American and (C) JHS African-American non-gout (control) groups.

**
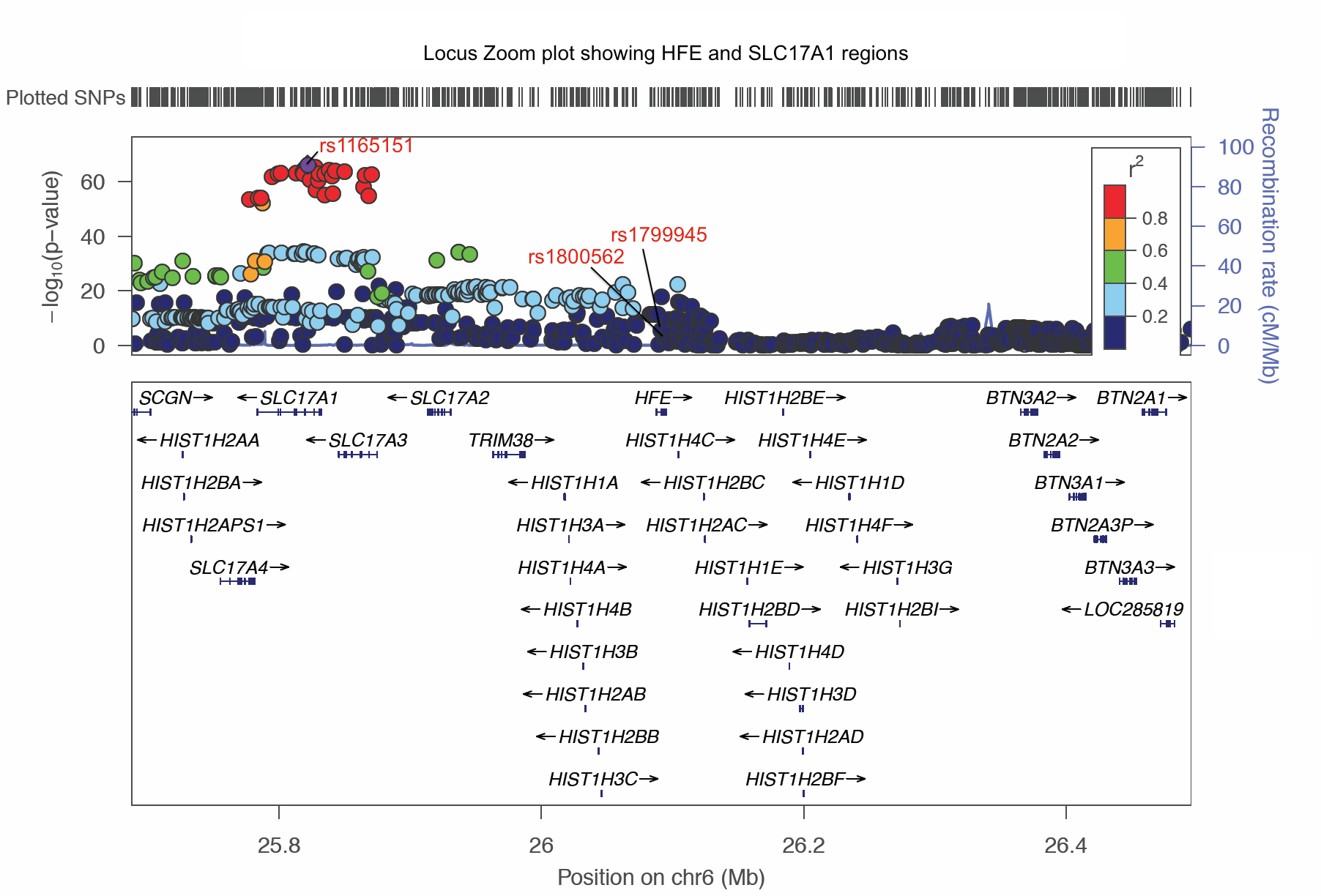
**

**Figure S3:** LocusZoom plot of association of the Chr6 *SLC17A1-A4 / HFE* locus with serum urate, generated from ref ^6^. The top associated

single-nucleotide polymorphism (SNP) is labeled, and other associated SNPs are colored according to strength of linkage disequilibrium (red = high;

purple = very low). The *HFE* SNPs are arrowed. –log10P for association with serum urate is on the left-hand y-axis.

**References**

1. Carpenter MA. Laboratory, reading center, and coordinating center data management methods in the Jackson Heart Study. *Am J Med Sci* 2004;3:131-44.

2. Li J, Lange LA, Duan Q, et al. Genome-wide admixture and association study of serum iron, ferritin, transferrin saturation and total iron binding capacity in African Americans. *Hum Mol Genet* 2015;15:572-81..

3. Fox ER, Benjamin EJ, Sarpong DF, et al. Epidemiology, heritability, and genetic linkage of C-reactive protein in African Americans (from the Jackson Heart Study). *Am J Cardiol* 2008;102:835-41.

4. Gunter EL, BL, Koncikowski, SM. Laboratory procedures used for the Third National Health and Nutrition Examination Survey (NHANES III), 1988–1994. In: Prevention USDOHAHSCfDCa, ed. NHANES III Reference Manuals and Reports. Hyattsville, MD, 1996:VII-R-1.

5. Benyamin B, Tonu E, Ried JS, et al. Novel loci affecting iron homeostasis and their effects in individuals at risk for hemochromatosis. *Nat Comm* 2014;5:4926.

6. Köttgen A, Albrecht E, Teumer A, et al. Genome-wide association analyses identify 18 new loci associated with serum urate concentrations. *Nat Genet* 2013;45:145-54.

7. Westra H-J, Peters MJ, Esko T, et al. Systematic identification of trans eQTLs as putative drivers of known disease associations. *Nat Genet* 2013;45:1238-43.

8. Caron L, Rousseau F, Gagnon É, et al. Cloning and functional characterization of a cation-Cl− cotransporter-interacting protein. *J Biol Chem* 2000;275:32027-36.

9. Markadieu N, Delpire E. Physiology and pathophysiology of SLC12A1/2 transporters. *Pflügers Arch* 2014;466:91-105.

10. Orlov SN, Koltsova SV, Kapilevich LV, et al. NKCC1 and NKCC2: The pathogenetic role of cation-chloride cotransporters in hypertension. *Genes Dis* 2015;2:186-96.

11. Wnuk M, Hlushchuk R, Janot M, et al. Podocyte EphB4 signaling helps recovery from glomerular injury. *Kidney Int* 2012;81:1212-25.

12. del Greco F, Foco L, Pichler I, et al. Serum iron level and kidney function: a Mendelian randomization study. *Nephrol Dial Transplant* 2017;32:273-8.

13. Zhao W, Wang L, Zhang M, et al. E3 ubiquitin ligase tripartite motif 38 negatively regulates TLR-mediated immune responses by proteasomal degradation of TNF receptor-associated factor 6 in macrophages. *J Immunol* 2012;188:2567-74.

14. Jeong J, Rao AU, Xu J, et al. The PRY/SPRY/B30. 2 Domain of Butyrophilin 1A1 (BTN1A1) Binds to Xanthine Oxidoreductase implications for the function of BTN1A1 in the mammary gland and other tissues. *J Biol Chem* 2009;284:22444-56.

15. Pérez-Magán E, de Lope ÁR, Ribalta T, et al. Differential expression profiling analyses identifies downregulation of 1p, 6q, and 14q genes and overexpression of 6p histone cluster 1 genes as markers of recurrence in meningiomas. *Neuro Oncol* 2010;12:1278-90.

16. Van Der Harst P, Zhang W, Leach IM, et al. Seventy-five genetic loci influencing the human red blood cell. *Nature* 2012;492:369-75.

17. Magoulas C, Fried M. Isolation and genomic analysis of the human surf-6 gene: a member of the Surfeit locus. Gene 2000;243:115-23.

18. Haslam DB, Baenziger JU. Expression cloning of Forssman glycolipid synthetase: a novel member of the histo-blood group ABO gene family. *Proc Natl Acad Sci U S A* 1996;93:10697-702.

19. Nai A, Pagani A, Silvestri L, et al. TMPRSS6 rs855791 modulates hepcidin transcription in vitro and serum hepcidin levels in normal individuals. *Blood* 2011;118:4459-62.

20. Pei S-N, Ma M-C, You H-L, et al. TMPRSS6 rs855791 polymorphism influences the susceptibility to iron deficiency anemia in women at reproductive age. *Int J Med Sci* 2014;**11**:614-9.

21. Du X, She E, Gelbart T, et al. The serine protease TMPRSS6 is required to sense iron deficiency. *Science* 2008;320:1088-92.

22. Camaschella C. Diagnosis and treatment of non-HFE-haemochromatosis. Disorders of iron homeostasis, erythrocytes, erythropoiesis. *Paris: ESH and Club du Globule Rouge et du Fer* 2006:467-73.

23. Altès A, Bach V, Ruiz A, et al. Does the SLC40A1 gene modify HFE-related haemochromatosis phenotypes? *Ann Hematol* 2009;88:341-5.

24. Moreno‐Carralero MI, Muñoz‐Muñoz JA, Cuadrado‐Grande N, et al. A novel mutation in the SLC40A1 gene associated with reduced iron export in vitro. *Am J Hematol* 2014;89:689-94.

25. Desuzinges-Mandon E, Arnaud O, Martinez L, et al. ABCG2 transports and transfers heme to albumin through its large extracellular loop. *J Biol Chem* 2010;285:33123-33.
